# Experimental evidence for the immediate impact of fertilization and irrigation upon the plant and invertebrate communities of mountain grasslands

**DOI:** 10.1002/ece3.1118

**Published:** 2014-05-28

**Authors:** Aline Andrey, Jean-Yves Humbert, Claire Pernollet, Raphaël Arlettaz

**Affiliations:** 1 Division of Conservation Biology, Institute of Ecology and Evolution, University of Bern3012, Bern, Switzerland; 2 Office National de la Chasse et de la Faune Sauvage, CNERA Avifaune MigratriceLa Tour du Valat, Le Sambuc, 13200, Arles, France; 3 Swiss Ornithological InstituteValais Field Station, Rue du Rhône 11, 1950, Sion, Switzerland

**Keywords:** Agriculture, arthropods, grassland management, hump-shaped model, liquid manure, vegetation heterogeneity

## Abstract

The response of montane and subalpine hay meadow plant and arthropod communities to the application of liquid manure and aerial irrigation – two novel, rapidly spreading management practices – remains poorly understood, which hampers the formulation of best practice management recommendations for both hay production and biodiversity preservation. In these nutrient-poor mountain grasslands, a moderate management regime could enhance overall conditions for biodiversity. This study experimentally assessed, at the site scale, among low-input montane and subalpine meadows, the short-term effects (1 year) of a moderate intensification (slurry fertilization: 26.7–53.3 kg N·ha^−1^·year^−1^; irrigation with sprinklers: 20 mm·week^−1^; singly or combined together) on plant species richness, vegetation structure, hay production, and arthropod abundance and biomass in the inner European Alps (Valais, SW Switzerland). Results show that (1) montane and subalpine hay meadow ecological communities respond very rapidly to an intensification of management practices; (2) on a short-term basis, a moderate intensification of very low-input hay meadows has positive effects on plant species richness, vegetation structure, hay production, and arthropod abundance and biomass; (3) vegetation structure is likely to be the key factor limiting arthropod abundance and biomass. Our ongoing experiments will in the longer term identify which level of management intensity achieves an optimal balance between biodiversity and hay production.

## Introduction

Numerous studies have documented that grassland management intensification alters biodiversity, leading to decline of plant and arthropod species richness and modifying plant traits as well as community structure (e.g., Marini et al. 2008; Riedener et al. 2013; Niu et al. 2014). Similarly, but on the other extreme of the grassland management intensity gradient, abandonment occurring in steep and less accessible mountain regions leads to forest encroachment and the disappearance of many open-habitat species (MacDonald et al. 2000; Tasser et al. 2007). However, alternatives to this dichotomous trend (agriculture intensification versus abandonment) exist in the form of an intermediate intensity of management in terms of mowing regime (e.g., Tonn and Briemle 2010; Bernhardt-Romermann et al. 2011), irrigation (Jeangros and Bertola 2000), and fertilization (e.g., Pauli et al. 2002; Bowman et al. 2006). This moderate management is likely to have conjugated positive effects on plant and invertebrate diversity, hay production, and forage nutritional quality. Different theories and factors can explain why an intermediate or moderate management intensity is likely to benefit grassland flora and fauna communities. For example, based on the hump-shaped species diversity curve of Grime (1973; see also Mittelbach et al. 2001), a moderate addition of resources should enhance plant species growth and richness. This phenomenon is especially expected in nutrient-poor montane and subalpine grasslands (Peter et al. 2009). In turn, an increase in plant growth will provide more food, space, and shelters for arthropods, boosting their abundances (e.g., Haddad et al. 2000; Perner et al. 2005; Dittrich and Helden 2011; Buri et al. 2013). Higher plant species richness not only provides more potential host plants for herbivores, but also greater horizontal and vertical vegetation structure complexity, which seems to be crucial to support higher diversity and abundance of arthropods (e.g., Brown et al. 1992; Morris 2000; Woodcock et al. 2009; Dittrich and Helden 2011). A more abundant arthropod community will promote higher trophic levels up to vertebrates through a cascading process (Hunter and Price 1992; Britschgi et al. 2006). In seminatural mountain meadows, the exact management practices that would permit decent hay production without degrading the functional integrity of the system remain unknown, thus meriting further investigation.

We launched a two-way factorial experiment on the short-, mid-, and long-term effects of fertilization and irrigation on plant and arthropod communities of montane and subalpine hay meadows of the inner European Alps (Valais, SW Switzerland). The main objective of this study is to document the short-term changes that occurred just 1 year after the onset of differential experimental management treatments. While end-user management recommendations will be based on the longer-term outputs of the study, thoroughly assessing the short-term effects clarifies the ecological mechanisms at play during the temporal process of grassland intensification. More specifically, we addressed two questions: (1) What are the short-term effects of fertilization and irrigation, considered separately and in combination, on plant species richness, vegetation structure, hay production, and arthropod abundance and biomass? and (2) what is the relationship between vegetation and arthropod parameters?

Plants and arthropods were hypothesized to respond differently to the fertilization and irrigation treatments in the short-term, that is, after just 1 year of experimental manipulation, partly because plants typically have a slower reaction time than animals to changes in environmental conditions (Mortimer et al. 1998; Cole et al. 2010). More specifically, we expected slight positive effects of fertilization on plant species richness and hay production (Grime 1973), while an increase in plant growth and richness was expected to increase vegetation structure, which would in turn promote arthropod populations (Woodcock et al. 2009). On the other hand, we predicted that irrigation would have no effect on plant species richness (Riedener et al. 2013), but still positive effects on arthropod abundance through an increased phytomass productivity and protection against dessication (Nielsen 1955). Fertilization was also predicted to increase herbivorous arthropod abundances, owing to an increase in plant tissue nitrogen content (Haddad et al. 2000; Dittrich and Helden 2011). However, due to a highly diverse plant species pool among all our meadows (given that they have been extensively managed over the past decades), a high ecological stability and resistance against the experimental treatments were expected in the short term (Tilman and Downing 1994), therefore translating into few contrasted effects.

## Materials and Methods

### Study sites

In 2010, twelve extensively managed montane and subalpine hay meadows were selected according to their management history. The meadows had to be managed extensively for at least the last 10 years with no or very low levels of fertilization (with solid manure only) and irrigation (terrestrial only), and only a single cut per year. Their homogeneous topography and their size were also considered (>4000 m^2^). The study sites were situated in the inner Alps (Valais, SW Switzerland) between 790 and 1740 m above sea level, encompassing a wide gradient of altitudes and ambient temperatures (Table 1). This region experiences a continental climate with cold and wet winters, and dry and hot summers.

**Table 1:** Description of the twelve study sites with altitude, exact coordinates, and quantity of fertilizer, that is, nitrogen (N), phosphorus (P), and potassium (K), applied per hectare per year. The fertilizer consisted of organic NPK pellets, and mineral K_2_O dissolved in water to reach the equivalent of standard-farm liquid manure

			Coordinates		Fertilizer applied [kg·ha^−1^·year^−1^]		
				
Site	Name	Altitude [m]	Latitude	Longitude	N	P	K
1	La Garde	980	46°3′45″N	7°8′35″E	40.0	33.3	133.3
2	Sembrancher	798	46°4′24″N	7°8′36″E	53.3	44.4	177.7
3	Orsières	1022	46°1′44″N	7°9′8″E	53.3	44.4	177.7
4	Vens	1373	46°5′7″N	7°7′24″E	40.0	33.3	133.3
5	Euseigne	1028	46°10′9″N	7°25′27″E	53.3	44.4	177.7
6	Eison	1768	46°9′18″N	7°28′10″E	26.7	22.3	89.0
7	St-Martin	1589	46°11′8″N	7°26′43″E	26.7	22.3	89.0
8	Grimentz	1738	46°11′22″N	7°34′35″E	26.7	22.3	89.0
9	Arbaz	1270	46°16′42″N	7°22′47″E	40.0	33.3	133.3
10	Icogne1	1200	46°17′56″N	7°26′31″E	40.0	33.3	133.3
11	Icogne2	880	46°17′6″N	7°26′10″E	53.3	44.4	177.7
12	Cordona	1153	46°19′45″N	7°33′8″E	40.0	33.3	133.3

### Design

**Figure 1:**
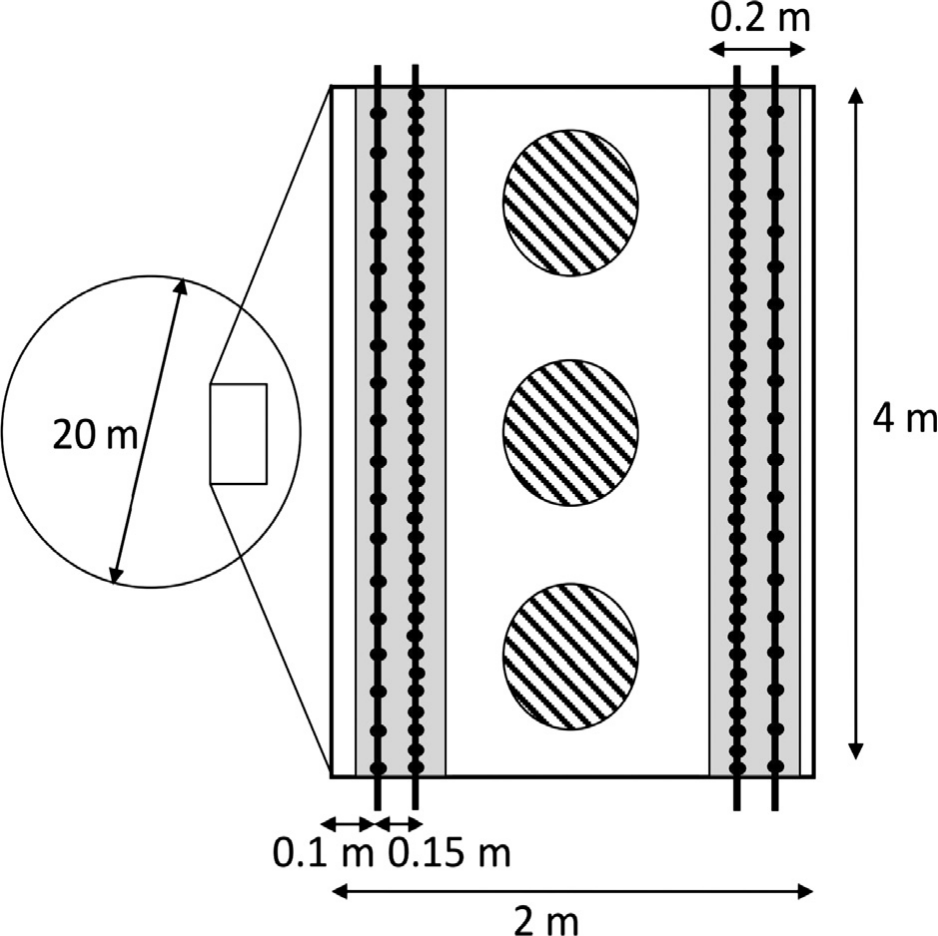
Experimental design. Four management treatments were applied at random onto 20-m-diameter circles delineated on each meadow. In each circle (excerpt), vegetation (*n* = 122 records per circle, black dots), hay production (gray strips), and arthropods (three dashed circles of 0.2 m^2^) were sampled.

A two-way full factorial design was applied in our experiments. At each study site, that is, in each meadow, four circular plots of 20 m in diameter were established with at least 5 m between plot boundaries. The different management treatments were randomly assigned to the four plots within a given meadow. The first plot served as a control (C-plot: neither irrigation nor fertilization). The second plot was only irrigated (I-plot) at regular time intervals with sprinklers. The third plot was only fertilized (F-plot) with liquid manure, and the fourth plot was irrigated and fertilized (I + F-plot). C-plots were cut once a year, which corresponds to local standards for extensively managed meadows, while I, F, and I + F-plots were cut twice a year. Although this discrepancy deviated the design from a purely speaking two-way full factorial design, it made agronomical sense; local farmers would not irrigate or fertilize their field without doing a second cut. Treatments I and I + F were irrigated weekly from mid-May to the beginning of September, except when heavy rainfall occurred (>20 mm over the previous week). Weekly sprinkler irrigation amounted to 20 mm of water column. The fertilizer consisted of organic dried manure NPK pellets (MEOC SA, 1906 Charrat, Switzerland), and mineral potassium oxide (K_2_O) dissolved in water to reach the equivalent of standard-farm liquid manure (Sinaj et al. 2009), consisting namely of 2.4 kg of usable nitrogen, 2 kg of phosphate (P_2_O_5_), and 8 kg of potassium oxide (K_2_O) per m^3^ of solution. 174, 262, or 349 l of liquid manure per plot, corresponding to, respectively, 26.7, 40.0, or 53.3 kg N·ha^−1^ year^−1^, were applied three times in August 2010, May 2011, and August 2011 (Table 1). The exact amount of manure applied at each site depended on the theoretical local hay production potential calculated using pre-experimental hay yield (when extensively managed) and site elevation, and it matched the local mid-intensive management norm recommended in Sinaj et al. (2009). In each plot, a 4 × 2 m permanent rectangle subplot was established at a distance of 4 m from plot center, randomly placed along the slope axis on the right or the left side of the plot. In each subplot, we measured plant species richness, vegetation structure, hay production, and abundance and biomass of arthropods (Fig. 1).

### Vegetation sampling

In 2011, plant species richness, vegetation structure, and hay production were assessed twice: once just before the first cut (from mid-June to end of July, at a similar vegetation stage, depending on altitude; hereafter referred to as July samples) and once just before the second cut (from August to September; hereafter August samples). Vegetation surveys were performed using the point quadrat method in order to obtain information on the vertical distribution of each plant species (Stampfli 1991). For that purpose, we developed an ad hoc device that consisted of a 4.10-m-long steel bar (supported by two tripods) that contained 41 holes distant of 10 cm (Appendix 1). Graduated metal sticks of 5 mm in diameter were inserted vertically into the holes. Each plant species touching the stick was recorded, and the height at which the plant touched the stick was noted. If the same species touched more than once a single stick, the maximal height was retained. The sampling device was positioned along each long side of the permanent rectangular subplot, first 10 cm and then 25 cm from the long edge (Fig. 1). We recorded contacts between plants and sticks at 20 and 41 holes (points) when the device was positioned at 10 cm and 25 cm from the edge, respectively. Altogether, we thus recorded 122 points in each plot. A modified Shannon–Wiener diversity index (Woodcock et al. 2009) was used to define the structure of the vegetation:


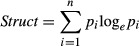


where *Struct* is the index for vegetation structure and *p*_*i*_ the proportion of the number of contacts with the stick at each height *i*, in each subplot, at each sampling session. Thus, greater structural complexity of the vegetation results in a higher value.

Just before each grass cut, hay production was estimated by clipping two strips of grass with an area of 0.2 × 4 m along each long edge of the permanent subplot at 6 cm above the ground, exactly where the vegetation relevés had been performed (Fig. 1). The two samples from the same subplot were then pooled together. The collected plant material was dried at 105°C during 72 h and then weighed (±0.1 g) in order to quantify hay production.

### Arthropod sampling

Arthropods were sampled using a suction sampler (Stihl SH 86 D; Stihl) equipped with a gauze sampling sack fixed inside the nozzle to collect arthropod items. This technique has been proved to be efficient for grassland vegetation-dwelling arthropods (Sanders and Entling 2011). All plots were sampled twice during the vegetation season, once before each grass cut. At each sampling session, three subsamples were collected at three regularly spaced locations in the middle of each permanent subplot (Fig. 1). Subsamples consisted of the vacuumed content of a metallic cylinder of 50 cm height and 50.5 cm diameter (0.2 m^2^ area) that was placed directly on the ground. The content of the gauze sampling sack was transferred into a sealed plastic bag stored at low temperature in an ice-cooled box. Sampling was undertaken between 11:00 and 17:00, only under dry vegetation conditions and with low or moderate wind. Arthropod specimens were then stored in the laboratory at −20°C before being classified in six main taxonomic groups: spiders, Auchenorrhyncha (i.e., plant- and leafhoppers), weevils, leaf beetles, ants, and others (other arthropods not belonging to the previous groups). The number of specimens was counted prior to drying the arthropods at 60° during 72 h. Finally, all arthropod groups stemming from one subsample were weighed (±0.1 mg). For statistical analyses, the three subsamples per plot were summed. Ants had to be discarded because suction trapping proved to be inefficient for sampling this group due to their massive local colonial aggregations.

### Statistical analysis

Treatment effects were analyzed with linear mixed-effects models (LMMs) using the *lmer* function from the *lme4* package for R (Bates et al. 2011). *P*-values and confidence intervals (CI) were computed with the *pvals.fnc* function from the *languageR* package using 100,000 Markov chain Monte Carlo iterations (Baayen 2011). Response variables were log-transformed plant species richness, vegetation structure, hay production, log-transformed arthropod abundance, and log-transformed arthropod biomass. As grass (Poaceae), legume (Fabaceae), and forb species may respond differently to the management treatments (e.g., Li et al. 2010), additional models on the relative cover of each functional group were run. Note that not all variables needed log-transformation prior to analysis to achieve normal distribution of residuals. The fixed effects were the treatments (C, I, F, or I + F) and the sampling sessions (July or August) which were added as a factor to take in account the fact that two measures were made per plot. For hay production, analyses were performed on the sum of the July and August (pooled samples). Thus, for this variable, fixed effects were limited to the treatments. The study sites (geographic replicates) were designated as a random effect. To better appraise differences between treatments, post hoc tests were performed using the function *relevel* of R to change the first reference level of the factor “treatment.”

In order to further understand the relationship between the vegetation and arthropod parameters, simple linear regressions were performed using the *lm* function (Crawley 2007). The log-transformed abundance and biomass of arthropods were fitted against plant species richness, vegetation structure (index *Struct*), and hay production. Finally, to test whether the variance in arthropod abundance and biomass (variance of the nontransformed raw data) changes with respect to vegetation structure, a homoscedasticity test (Bartlett's test) was conducted between the values obtained from the first and the third quantiles of *Struct* (Crawley 2007). Thus, a significant *P*-value would indicate that with low vegetation structure, there are only few arthropods, while with a higher vegetation structure, it is possible to have either few or many arthropods (see Fig. 4). In other words, this value indicates whether vegetation structure limits arthropod abundance and/or biomass. All statistical tests were performed using R version 2.15.3 (R Core Team 2013).

## Results

### Effects of irrigation and fertilization on the vegetation

**Figure 2:**
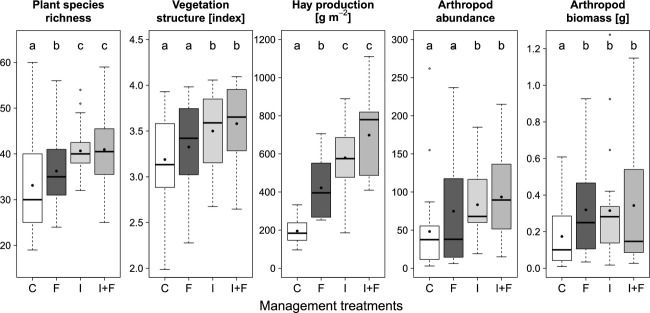
Responses of the vegetation (plant species richness, vegetation structure and hay production) and arthropod (abundance and dry biomass) variables to the different management treatments. Bold lines represent medians, solid points the means, boxes the first and third quantiles. Different letters indicate significant differences among treatments at an alpha rejection value set to 0.05. Treatments abbreviations are as follows: (C) control; (I) irrigated, (F) fertilized, and (I + F) irrigated and fertilized.

In total, 194 plant species belonging to 34 families were identified during the two sampling sessions across all meadows (see Appendix 2 for a complete list of the plant species recorded). F-plots, I-plots, and I + F-plots harbored significantly more plant species than C-plots (Fig. 2; and Table A3.1 in Appendix 3 for related model outputs). Moreover, irrigated plots (I and I + F) had significantly higher species richness than F-plots, but treatment I + F was not different from I. Irrigated plots exhibited a higher vegetation structure (index *Struct*) than C-plots and F-plots, while treatment F did not differ from C. The greatest vegetation structure was measured in July and the lowest in August; this pattern was consistent across all treatments. Annual hay production (sum of both sampling sessions) ranged from 96.5 to 1111 g·m^−2^ across all plots. It was approximately three times higher in the irrigated plots compared with C-plots, but I + F treatment did not differ from treatment I. Fertilization (F) had a lower effect compared with irrigation but still gave a significantly higher hay production than C.

**Figure 3:**
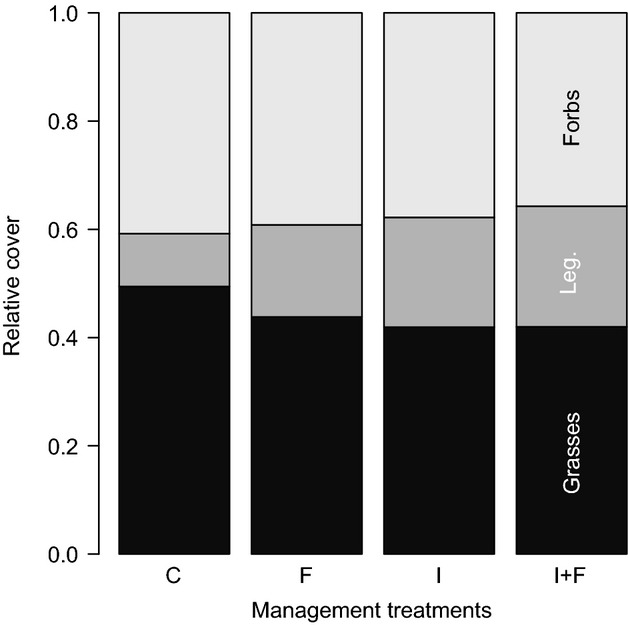
Responses of relative cover of grass (dark-gray), legume (mid-gray), and forb (light-gray) species to the different management treatments. Model outputs (including estimates, CIs, and *P*-values) are provided in Table A3.2 in Appendix 3. For treatment abbreviations, see legend of Fig. 2.

Relative cover of grasses decreased in I, F, and I + F-plots compared with the control plots, while legumes increased their cover (Fig. 3). Relative changes were all significant at a *P* < 0.01 level (see Table A3.2 in Appendix 3 for exact values of models outputs). Forb species cover did not differ among treatments except I + F that had significantly less cover than C (*P* = 0.011).

### Effects of irrigation and fertilization on the arthropods

In total, 7198 arthropods (ants excluded) were collected across all replicates (3923 in July and 3275 in August). The samples included *n* individuals of the following taxa: 629 spiders (Araneae), 1869 plant- and leafhoppers (Hemiptera: Auchenorrhyncha), 562 weevils (Coleoptera: Curculionidae), 587 leaf beetles (Coleoptera: Chrysomelidae), and 3551 others. Abundance of arthropods in I-plots and I + F-plots were significantly higher than in C-plots and F-plots (Fig. 2; and Table A3.1 in Appendix 3 for related model outputs). Treatment F did not deliver a higher abundance of arthropods compared with treatment C. The only significant differences within a single arthropod group were for plant- and leafhoppers where in I + F-plots, there were more individuals compared with C-plots (MCMC mean = 0.890, 95% CI = 0.281–1.511, *P* MCMC = 0.005) and to F-plots (MCMC mean = 0.766, 95% CI = 0.161–1.385, *P* MCMC = 0.015). For spiders, abundance in I + F-plots was marginally significantly higher than in C-plots (MCMC mean = 0.375, 95% CI = −0.021–0.759, *P* MCMC = 0.060), while no differences were detected between I-plots and F-plots, on one side, and C-plots, on the other side.

In total, 26.92 g dry weight of arthropods was collected across all replicates (17.13 g in July and 9.79 g in August). The samples (excluding ants) included the following taxa: 1.856 g of spiders, 2.705 g of plant- and leafhoppers, 0.766 g of weevils, 0.458 g of leaf beetles, and 21.130 g for others. All treatments affected positively the biomass of arthropods (Fig. 2; and Table A3.1 in Appendix 3). The biomass of plant- and leafhoppers was significantly higher in I + F-plots than in the C-plots (MCMC mean = 0.019, 95% CI = 0.001–0.037, *P* MCMC = 0.038), while there were no significant biomass differences between treatments and controls in another arthropod taxonomic group.

### Relationships between arthropods and vegetation

**Figure 4:**
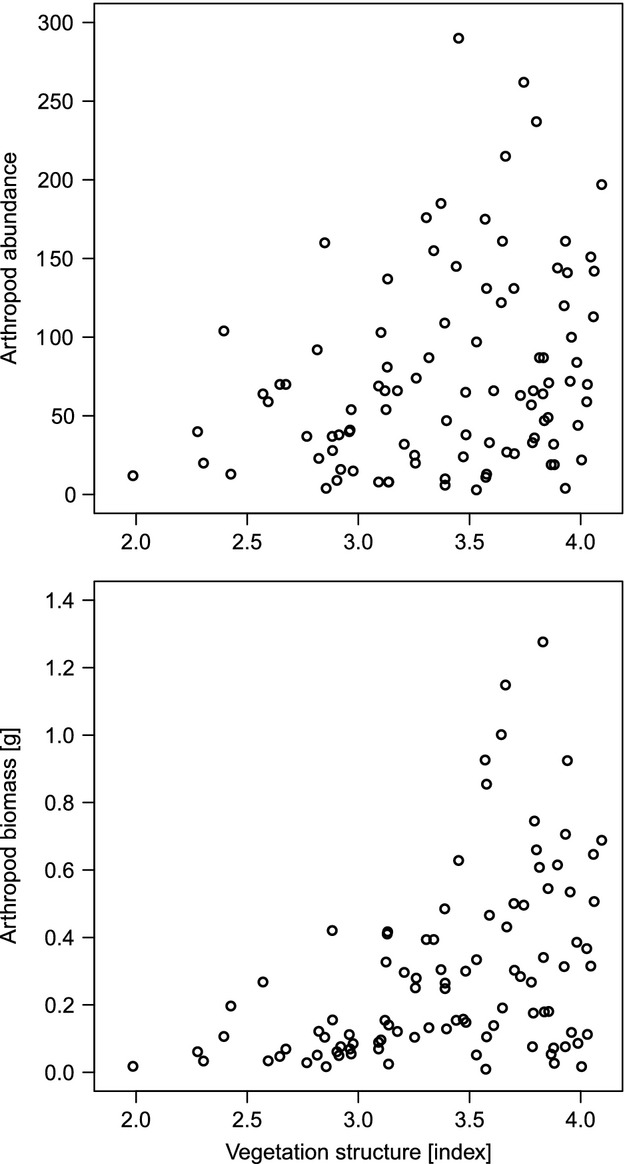
Relationships between arthropod abundance and biomass versus vegetation structure (index *Struct*). Greater the structure of the vegetation, higher the *Struct* index.

The total abundance of arthropods was positively linked to hay production (estimate = 2.60·10^−3^ *t* = 4.767, *P* < 0.001; adjusted *R*^2^ = 0.186, i.e. 18.6% explained variance), plant species richness (estimate = 6.79·10^−2^ *t* = 6.696; *P* < 0.001, *R*^2^ = 0.316), and vegetation structure (estimate = 0.572, *t* = 2.752, *P* = 0.007, *R*^2^ = 0.065). The variance in arthropod biomass was explained in about the same order of magnitude by hay production (estimate = 2.905·10^−3^ *t* = 5.085, *P* < 0.001, *R*^2^ = 0.207), plant species richness (estimate = 5.580·10^−2^ *t* = 4.747, *P* < 0.001, *R*^2^ = 0.185), and vegetation structure (estimate = 1.049, *t* = 5.182, *P* < 0.001, adjusted *R*^2^ = 0.214). Note that estimates are on the log scale. Regarding the analyses about whether vegetation structure limits arthropods, for both arthropod abundance (Bartlett's *K*^2^ = 6.933, df = 1, *P* = 0.008) and biomass (Bartlett's *K*^2^ = 23.145, df = 1, *P* < 0.001), Bartlett's test showed a greater variance at the third than at the first quantile of vegetation structure (Fig. 4).

## Discussion

This study shows that among low-input montane and subalpine hay meadows, plant species richness, vegetation structure, hay production as well as arthropod abundance and biomass all immediately and positively react to moderate experimental fertilization and irrigation. It should be noted, however, that the starting conditions in our study meadows were typical of the traditional, extremely extensive management practices that have been prevailing for centuries in the inner Alps, with very low fertilizer application and limited terrestrial irrigation. It is thus not totally surprising that our experimental treatments improved both biodiversity and hay yield in the very short term. These traditional grasslands typically are poor in nitrophilous species with specialized taxa present due to a very constraining edaphic context and watering regime (Peter et al. 2009). The speed at which these changes operated in response to intensification was, however, unexpectedly rapid. A powerful advantage of our full block design approach is certainly that it allows a direct comparison of the effects of both irrigation and fertilization, which were either separated or conjugated, upon meadowland ecological communities regardless of other potentially confounding abiotic factors such as altitude, exposition, or soil properties.

### Effects of fertilization and irrigation on the vegetation

Fertilizing with liquid manure and watering with sprinklers are two modern, currently spreading management practices, even in remote areas of the Alps (Riedener et al. 2013). Our treatments thus mimic the trends of modern agriculture in these areas. Although we had predicted slower effects on plant species richness, basing our predictions on the dynamics observed in most long-term studies in alpine and arctic regions (e.g., Carlen et al. 1998; Yang et al. 2011), our findings are in accordance with the predictions of the hump-shaped model of plant diversity (Grime 1973; Mittelbach et al. 2001). This model stipulates that an intermediate level of intensification must support a higher plant species richness than low- or high-input systems. Yet, we cannot exclude, given that we measured effects just 1 year after the onset of the experimental treatments, that abiotic factors, such as interannual weather variation, might have interacted with the treatment effects, amplifying the signal (Walker et al. 1994). What is certain, however, is that no plant community would ever reach an equilibrium after just 1 year of this management (Yang et al. 2011). Hence, a short-term, moderate intensification as applied here may indeed promote high plant species richness because it rapidly offers favorable conditions to nitrophilous and mesophilous species that are normally absent on nutrient-poor and dry soils. Some of the original plant species pool consisting of heliophilous species, tolerant to reduced nutrients and water supply but particularly intolerant to intensification and shade, may actually have persisted in the community merely because they were already extant. This suggests the possibility of a short-term coexistence of plants with different life-history traits and varied ecological requirements (Bowman et al. 2006). In the mid- and long run, however, one would expect that interspecific competition for resources such as light will especially increase among some species. Species exhibiting characteristics such as low growth rate could become progressively disadvantaged and possibly decline to local extinction (Rajaniemi 2002; Hautier et al. 2009).

Irrigating and fertilizing increased the relative cover of legumes, which appears to be mostly at the expense of the cover of grasses. While this seems in contradiction with most grassland fertilization studies that found the reverse pattern regarding their biomasses (e.g. DiTommaso and Aarssen 1989; Carlen et al. 1998; Li et al. 2010), it must be stressed that relative cover does not necessarily correlate with biomass, especially when comparing grasses that grow tall and thin with legumes that tend to grow wider. In addition, fertilization studies that found positive effects of intensification on grasses and negative effects on legumes usually applied mineral fertilizers, while the application of organic fertilizers is known to have slightly different influences, typically favoring legume species (e.g., Vintu et al. 2011).

In contradiction to our prediction that fertilization would have a positive short-term effect on all vegetation parameters, addition of liquid manure alone did not increase vegetation structure, while the combination of fertilization and irrigation did not elicit a greater response from vegetation parameters than did irrigation alone. This indicates that in the short term, application of fertilizer (only) might enhance the sensitivity of the vegetation to water stress (Huston 1997) or that our meadows were more likely to be limited by water supply than nitrogen supply. Indeed, the climatic context in the inner Alps is characterized by its dryness (Central Valais, around Sion-Visp, is the pole of xericity in the whole Alpine massif, with ca 500 mm annual precipitation), with even April-June 2011 slightly drier than interannual average (94 mm vs. 136 mm mean rainfall during 2006–2010 in Sion; MeteoSwiss). Plant nutrient uptake may also have been improved by water addition thus enhancing plant growth (Davis et al. 2000). Future vegetation surveys in the same study meadows will enable disentangling climatic from agronomic effects, while characterizing mid- and longer-term changes in plant communities.

### Effects of fertilization and irrigation on arthropod communities

Irrigation in turn had a positive effect on arthropod species richness, as predicted. This indicates that water might be a limiting factor for arthropods (e.g., intolerance to desiccation; Nielsen 1955), or that there is an indirect effect mediated via plants onto arthropods. In contrast, fertilization per se led to no discernible effect on arthropods, corroborating previous findings in comparable montane ecosystems (Grandchamp et al. 2005). The less complex vegetation structure achieved via fertilization alone compared with irrigation means that the offer of microhabitats and the resulting ecological niche opportunities are less favorable when only fertilization is augmented (Reid and Hochuli 2007). Irrigation and fertilization were also expected to increase the rate of herbivory, that is, the abundance of plant- and leafhoppers, and as a result increase the abundance of their predators such as spiders (Kirchner 1977). However, only plant- and leafhoppers showed a numeric response to irrigation and fertilization suggesting that a steady state had not been achieved with no discernible effects being propagated to the upper trophic levels along the food chain at this stage. It is also important to note that a much smaller sample size for predator taxa than for prey taxa could have blurred the pattern due to lower statistical power.

### Relationships between arthropods and vegetation

Vegetation parameters such as plant species richness, plant biomass, and vegetation structure all influence arthropod community to some extent (Knops et al. 1999; Haddad et al. 2000). There is still an ongoing debate about which factor has the greatest impact on arthropods (Perner et al. 2005), but recent studies have pointed out that vegetation structure might be the crux (Woodcock et al. 2009; Dittrich and Helden 2011). Our analyses show that all vegetation parameters influence arthropods to a certain degree. However, neither plant species richness (31.6% of explained variance for abundance/18.5% for biomass) nor hay production (18.6%/20.7%) or vegetation structure (6.5%/21.4%) individually accurately predicted arthropod abundance and biomass. This seems to contradict the view that vegetation structure is a key factor. However, there is evidence that vegetation structure did profoundly influence the number of arthropods in our meadows (Fig. 4), yet vegetation structure is more likely to act as a limiting than a predictive factor. Indeed, at low vegetation structure, low arthropod abundance and biomass always prevail, whereas at high vegetation structural diversity, arthropod abundance and biomass can either be low or high. This pattern is in line with the predictions of the habitat heterogeneity hypothesis (Brown et al. 1992). A higher entanglement of plant above-ground parts can increase the mobility of grass-dwelling arthropods (Randlkofer et al. 2009) through better vertical and horizontal connectivity while it offers a broader palette of ecological niches (Duffey 1962). Thus, if complex vegetation structure is a *sine qua non* condition for high arthropod abundance and biomass, it does not guarantee it. It is likely that source populations must exist in the surrounding matrix to colonize any newly emerging, highly structured vegetation patches. Moreover, new detrimental factors generated by high vegetation structure might also obliterate the ability of arthropod populations to develop, such as microclimatic conditions that adversely affect some taxa (increase moisture or shade) or altered diffusion of plant volatiles that hampers resource location (e.g. Van Wingerden et al. 1992; Finch and Collier 2000; Després et al. 2007).

## Conclusion

Although plant community stability was likely not achieved after just 1 year of experimental fertilization and irrigation, our findings demonstrate that on a short-term basis, a moderate level of intensification positively affects biodiversity and hay production of low-input, extensively managed montane and subalpine meadows. Tremendous land-use changes steadily affect mountainous regions, leading either to abandonment of marginal grasslands or to intensification of fields accessible to machinery (Tasser et al. 2007). This rather dichotomous trend should be reversed, which calls for more intermediate management practices if one wants to concomitantly promote grassland biodiversity and acceptable agricultural revenue. Although this short-term study only provides insights into the mechanism of intensification within upland grasslands, the continuation of our experiments will deliver detailed prescriptions in the mid term for optimizing slurry fertilization and aerial irrigation so as to achieve the best possible compromise between hay production, biodiversity preservation, and ecosystem functioning among montane and subalpine hay meadows.

## Appendix 2: A complete list of the plant species identified during the two sampling sessions across all treatments in all meadows

**Table A2.1:** In total, 194 plant species belonging to 34 families were identified during the two sampling sessions across all meadows

Plant species name	Family	Plant species name	Family
Achillea millefolium l.	Asteraceae	Crepis conyzifolia (Gouan)	Asteraceae
Acinos alpinus (l.) Moench	Lamiaceae	Crepis pyrenaica (l.) Greuter	Asteraceae
Agrimonia eupatoria l.	Rosaceae	Crocus albiflorus Kit.	Iridaceae
Agrostis capillaris l.	Poaceae	Cynosurus cristatus l.	Poaceae
Agrostis stolonifera l.	Poaceae	Dactylis glomerata l.	Poaceae
Ajuga pyramidalis l.	Lamiaceae	Dactylorhiza fuchsii (Druce) sod	Orchidaceae
Ajuga reptans l.	Lamiaceae	Descampsia sp	Poaceae
Alchemilla vulgaris aggr.	Rosaceae	Elymus repens (l.) Gould.	Poaceae
Allium oleraceum l.	Liliaceae	Erucastrum nastrurtiifolium	Brassicaceae
Anthericum ramosum l.	Liliaceae	Euphorbia cyparissias l.	Euphorbiaceae
Anthoxanthum odoratum l.	Poaceae	Euphorbia verrucosa l.	Euphorbiaceae
Anthriscus sylvestris (l.) Hoffm.	Apiaceae	Euphrasia rostkoviana aggr.	Scrophulaceae
Anthyllis vulneraria l.	Fabaceae	festuca arundinacea schreb.	Poaceae
Arabis ciliata Clairv.	Brassicaceae	festuca ovina l.	Poaceae
Arabis hirsuta (l.) scop.	Brassicaceae	festuca pratensis Huds.	Poaceae
Arenaria serpyllifolia l.	Caryophyllaceae	festuca rubra l.	Poaceae
Arrhenatherum elatius (l.)	Poaceae	festuca valesiaca Gaudin	Poaceae
Asperula cynanchica l.	Rubiaceae	filipendula vulgaris Moench	Rosaceae
Avenella flexuosa (l.) Drejer	Poaceae	Galium anisophyllum Vill.	Rubiaceae
Botrychium lunaria (l.) sw.	Ophiolglossaceae	Galium boreale l.	Rubiaceae
Brachypodium pinnatum (l.)	Poaceae	Galium mollugo aggr.	Rubiaceae
Briza media l.	Poaceae	Galium pumilum Murray	Rubiaceae
Bromus erectus Huds.	Poaceae	Galium verum l.	Rubiaceae
Bunium bulbocastanum l.	Apiaceae	Gentiana acaulis l.	Gentianacees
Campanula glomerata l.	Campanulaceae	Gentiana campestris l.	Gentianacees
Campanula rhomboidalis l.	Campanulaceae	Gentiana verna l.	Gentianacees
Campanula rotundifolia l.	Campanulaceae	Geranium sanguineum l.	Geraniaceae
Campanula scheuchzeri Vill.	Campanulaceae	Geranium sylvaticum l.	Geraniaceae
Cardamina hirsuta	Brassicaceae	Geum montanum l.	Rosaceae
Carex caryophyllea latourr.	Cyperaceae	Gymnadenia conopsea (l.) r. Br.	Orchidaceae
Carex flacca schreb.	Cyperaceae	Helianthemum nummularium (l.) Mill.	Cistaceae
Carex montana l.	Cyperaceae	Helictotrichon pubescens (Huds.) Pilg.	Poaceae
Carex ornithopoda Willd.	Cyperaceae	Hepatica nobilis schreb.	Renonculaceae
Carex pallescens l.	Cyperaceae	Heracleum sphondylium l.	Apiaceae
Carex sempervirens Vill.	Cyperaceae	Hieracium murorum aggr.	Asteraceae
Carlina acaulis l.	Asteraceae	Hieracium piloselloides Vill.	Asteraceae
Carum carvi l.	Apiaceae	Hippocrepis comosa l.	Fabaceae
Centaurea jacea l.	Asteraceae	Hypericum perforatum l.	HypEricaceae
Centaurea scabiosa l.	Asteraceae	Hypochoeris maculata l.	Asteraceae
Cerastium arvense l.	Caryophyllaceae	Inula salicina l.	Asteraceae
Cerastium fontanum	Caryophyllaceae	Knautia arvensis (l.) Coult.	Dipsacaceae
Chaerophyllum hirsutum l.	Apiaceae	Knautia dipsacifolia Kreutzer	Dipsacaceae
Cirsium acaule scop.	Asteraceae	Koeleria pyramidata (lam.) P. Beauv.	Poaceae
Cirsium arvense (l.) scop.	Asteraceae	laserpitium latifolium l.	Apiaceae
Clinopodium vulgare l.	Lamiaceae	laserpitium siler l.	Apiaceae
Colchicum alpinum DC.	Liliaceae	lathyrus pratensis l.	Fabaceae
Colchicum autumnale l.	Liliaceae	leontodon hispidus l.	Asteraceae
Crepis aurea (l.) Cass.	Asteraceae	leucanthemum vulgare aggr.r	Asteraceae
Crepis biennis l.	Asteraceae	linaria vulgaris Mill.	Scrophulaceae
linum catharticum l.	Linaceae	Prunella vulgaris l.	Lamiaceae
listera ovata (l.) r. Br.	Orchidaceae	Pulmonaria australis (Murr)	Lamiaceae
lolium perenne l.	Poaceae	Pulsatilla alpina (l.) Delarbre	Renonculaceae
lotus corniculatus l.	Fabaceae	ranunculus acris l.	Renonculaceae
luzula campestris (l.) DC.	Joncaceae	ranunculus bulbosus l.	Renonculaceae
luzula nivea (l.) DC.	Joncaceae	ranunculus montanus aggr.	Renonculaceae
luzula sylvatica aggr.	Joncaceae	ranunculus tuberosus lapeyr.	Renonculaceae
Medicago lupulina l.	Fabaceae	rhinanthus alectorolophus (scop.)	Scrophulaceae
Molinia arundinacea schrank	Poaceae	rosa pendulina l.	Rosaceae
Molinia caerulea (l.) Moench	Poaceae	rubus caesius l.	Rosaceae
Myosotis arvensis Hill.	Boraginaceae	rumex acetosa l.	Polygonaceae
Myosotis sylvatica Hoffm.	Boraginaceae	salvia pratensis l.	Lamiaceae
Nardus stricta l.	Poaceae	sanguisorba minor scop.	Rosaceae
Onobrychis viciifolia scop.	Fabaceae	sanguisorba officinalis l.	Rosaceae
Ononis repens l.	Fabaceae	scabiosa columbaria l.	Dipsacaceae
Ononis spinosa l.	Fabaceae	securigera varia (l.) lassen	Fabaceae
Paradisea liliastrum (l.) Bertol.	Liliaceae	selaginella selaginoides (l.)	Selaginellaceae
Pastinaca sativa l.	Apiaceae	sesleria caerulea (l.) Ard.	Poaceae
Peucedanum oreoselinum (l.)	Apiaceae	silene nutans l.	Caryophyllaceae
Phleum alpinum l.	Poaceae	silene vulgaris (Moench) Garcke	Caryophyllaceae
Phleum pratense l.	Poaceae	soldanella alpina l.	Primulaceae
Phyteuma betonicifolium Vill.	Campanulaceae	stachys recta l.	Lamiaceae
Phyteuma orbiculare l.	Campanulaceae	Taraxacum officinale aggr.	Asteraceae
Phyteuma spicatum l.	Campanulaceae	Thalictrum minus aggr.	Renonculaceae
Picris hieracioides l.	Asteraceae	Thesium alpinum l.	Santalaceae
Pimpinella saxifraga l.	Apiaceae	Thesium pyrenaicum Pourr.	Santalaceae
Plantago atrata Hoppe	Plantaginaceae	Thymus serpyllum aggr.	Lamiaceae
Plantago lanceolata l.	Plantaginaceae	Tragopogon pratensis l.	Asteraceae
Plantago media l.	Plantaginaceae	Trifolium alpestre l.	Fabaceae
Poa alpina l.	Poaceae	Trifolium badium schreb.	Fabaceae
Poa bulbosa l.	Poaceae	Trifolium dubium sibth.	Fabaceae
Poa pratensis l.	Poaceae	Trifolium medium l.	Fabaceae
Poa trivialis l.	Poaceae	Trifolium montanum l.	Fabaceae
Polygala alpestris rchb.	Polygalceae	Trifolium pratense l.	Fabaceae
Polygala chamaebuxus l.	Polygalceae	Trifolium repens l.	Fabaceae
Polygala comosa schkuhr	Polygalceae	Trisetum flavescens (l.) P. Beauv.	Poaceae
Polygala sp.	Polygalceae	Trollius europaeus l.	Renonculaceae
Polygala vulgaris l.	Polygalceae	Vaccinium myrtillus l.	Ericaceae
Polygonatum odoratum	Liliaceae	Verbascum nigrum l.	Scrophulaceae
Polygonum viviparum l.	Polygonaceae	Veronica arvensis l.	Scrophulaceae
Potentilla aurea l.	Rosaceae	Veronica chamaedrys l.	Scrophulaceae
Potentilla crantzii fritsch	Rosaceae	Veronica teucrium l.	Scrophulaceae
Potentilla erecta (l.) raeusch.	Rosaceae	Vicia cracca l.	Fabaceae
Potentilla pusilla Hostr	Rosaceae	Vicia sativa l.	Fabaceae
Potentilla rupestris l.	Rosaceae	Vicia sepium l.	Fabaceae
Potentilla thuringiaca link	Rosaceae	Viola hirta l.	Violaceae
Primula veris l.	Primulaceae	Viola rupestris f. W. schmidt	Violaceae
Prunella grandiflora (l.) scholler	Lamiaceae	Viola tricolor l.	Violaceae

## Appendix 3: Outputs of the linear mixed-effects models (LMMs) carried out on: (1) the effects of fertilization and irrigation on plant species richness, vegetation structure, hay production, arthropod abundance and biomass; and (2) the effects of fertilization and irrigation on the relative cover of grass, legume, and forb species. Table A3.1 refers to figure 2, and Table A3.2 refers to figure 3

**Table A3.1.** Results of the linear mixed-effects models (LMMs) carried out on the effects of fertilization and irrigation on plant species richness, vegetation structure, hay production, arthropod abundance and biomass. Table refers to Fig. 2 in the article. The fixed factors were the experimental treatments (with four levels: C = control plots; F = fertilized; I = irrigated; I + F = irrigation and fertilization combined) and the sampling sessions (two levels: July and August). Random factor were the experimental study sites. *P*-values and 95% confidence intervals (CI) were computed with 100,000 Markov chain Monte Carlo (MCMC) iterations. MCMC mean parameter estimates (differences between expected mean densities) are given for the paired treatments comparisons, and significant contrasts are highlighted in bold

Response variable and comparison	MCMC mean	Lower 95% CI	Upper 95% CI	MCMC *P*-value
Plant species richness (log scale)				
F vs. C	0.109	0.016	0.205	**0.023**
I vs. C	0.240	0.145	0.333	**<0.001**
I + F vs. C	0.236	0.144	0.331	**<0.001**
I vs. F	0.130	0.035	0.223	**0.007**
I + F vs. F	0.127	0.033	0.221	**0.009**
I + F vs. I	−0.003	−0.097	0.092	0.947
Structure of vegetation (index)				
F vs. C	0.136	−0.001	0.272	0.051
I vs. C	0.311	0.176	0.450	**<0.001**
I + F vs. C	0.392	0.255	0.529	**<0.001**
I vs. F	0.175	0.039	0.311	**0.012**
I + F vs. F	0.256	0.121	0.395	0.001
I + F vs. I	0.081	−0.054	0.219	0.247
Hay production [g·m^−2^]				
F vs. C	226.8	101.1	352.5	**0.001**
I vs. C	384.4	262.6	514.1	**<0.001**
I + F vs. C	503.2	379.7	630.8	**<0.001**
I vs. F	157.6	29.0	280.2	**0.015**
I + F vs. F	276.7	150.2	400.7	**<0.001**
I + F vs. I	118.8	−7.2	245.6	0.065
Arthropod abundance (log scale)				
F vs. C	0.403	−0.039	0.845	0.072
I vs. C	0.935	0.497	1.378	**<0.001**
I + F vs. C	1.014	0.579	1.452	**<0.001**
I vs. F	0.534	0.087	0.966	**0.018**
I + F vs. F	0.612	0.164	1.044	**0.006**
I + F vs. I	0.077	−0.365	0.514	0.730
Arthropod biomass [g] (log scale)				
F vs. C	0.829	0.327	1.303	**0.001**
I vs. C	0.824	0.325	1.306	**0.001**
I + F vs. C	0.734	0.237	1.219	**0.004**
I vs. F	−0.005	−0.501	0.477	0.983
I + F vs. F	−0.094	−0.579	0.397	0.706
I + F vs. I	−0.091	−0.587	0.389	0.716

**Table A3.2:** Results of the linear mixed effects models (LMMs) carried out on the effects of fertilization and irrigation on the relative cover of grass, legume and forb species. Table refers to Fig. 3 in the article. The fixed factors were the experimental treatments (with four levels: C = control plots; F = fertilized; I = irrigated; I+F = irrigation and fertilization combined) and the sampling sessions (two levels: July and August). Random factors were the experimental study sites. *P*-values and 95% confidence intervals (CI) were computed with 100,000 Markov chain Monte Carlo (MCMC) iterations. MCMC mean parameter estimates (differences between expected mean densities) are given for the paired treatments comparisons and significant contrasts are highlighted in bold

Response variable and comparison	MCMC mean	Lower 95% CI	Upper 95% CI	MCMC *P*-value
Grasses (Poaceae)				
F vs. C	−0.056	−0.099	−0.014	**0.009**
I vs. C	−0.075	−0.117	−0.033	**0.001**
I + F vs. C	−0.075	−0.116	−0.032	**0.001**
I vs. F	−0.019	−0.061	0.023	0.380
I + F vs. F	−0.018	−0.059	0.024	0.390
I + F vs. I	0.001	−0.042	0.043	0.974
Legumes (Fabaceae)				
F vs. C	0.073	0.037	0.108	**<0.001**
I vs. C	0.105	0.070	0.140	**<0.001**
I + F vs. C	0.125	0.091	0.162	**<0.001**
I vs. F	0.033	−0.003	0.068	0.070
I + F vs. F	0.053	0.018	0.088	**0.004**
I + F vs. I	0.020	−0.015	0.055	0.261
Forbs				
F vs. C	−0.016	−0.055	0.024	0.415
I vs. C	−0.030	−0.070	0.009	0.131
I + F vs. C	−0.051	−0.090	−0.012	**0.011**
I vs. F	−0.014	−0.054	0.025	0.479
I + F vs. F	−0.035	−0.074	0.005	0.083
I + F vs. I	−0.021	−0.059	0.020	0.302
